# Practical Considerations for Cardiac Electronic Devices Reimplantation Following Transvenous Lead Extraction Due to Related Endocarditis

**DOI:** 10.3390/jcm12216908

**Published:** 2023-11-02

**Authors:** Hussam Ali, Sara Foresti, Guido De Ambroggi, Riccardo Cappato, Pierpaolo Lupo

**Affiliations:** Arrhythmia & Electrophysiology Centre, IRCCS MultiMedica, 20099 Sesto San Giovanni, Italy; sara.foresti@multimedica.it (S.F.); guido.deambroggi@multimedica.it (G.D.A.); riccardo.cappato@multimedica.it (R.C.); pierpaolo.lupo@multimedica.it (P.L.)

**Keywords:** reimplantation, lead extraction, infective endocarditis, cardiac implantable electronic devices discipline

## Abstract

Despite progress in implantation technology and prophylactic measures, infection complications related to cardiac implantable electronic devices (CIED) are still a major concern with negative impacts on patient outcomes and the health system’s resources. Infective endocarditis (IE) represents one of the most threatening CIED-related infections associated with high mortality rates and requires prompt diagnosis and management. Transvenous lead extraction (TLE), combined with prolonged antibiotic therapy, has been validated as an effective approach to treat patients with CIED-related IE. Though early complete removal is undoubtedly recommended for CIED-related IE or systemic infection, device reimplantation still represents a clinical challenge in these patients at high risk of reinfection, with many gaps in the current knowledge and international guidelines. Based on the available literature data and authors’ experience, this review aims to address the practical and clinical considerations regarding CIED reimplantation following lead extraction for related IE, focusing on the reassessment of CIED indication, procedure timing, and the reimplanted CIED type and site. A tailored, multidisciplinary approach involving clinical cardiologists, electrophysiologists, cardiac imaging experts, cardiac surgeons, and infectious disease specialists is crucial to optimize these patients’ management and clinical outcomes.

## 1. Introduction

Through the last decades, the number of cardiac implantable electronic devices (CIED), including the pacemaker (PM), implantable cardioverter-defibrillator (ICD), and cardiac resynchronization therapy (CRT), has been increasing worldwide, involving more vulnerable patient cohorts with multiple comorbidities. Therefore, despite progress in implantation technology and prophylactic measures, the incidence of CIED infections is still relatively high, with substantial negative impacts on patients’ outcomes and the health system’s resources [1,2].

Aiming to simplify the implantation procedure and reduce the related infections, novel device technologies have been introduced into clinical practice with favorable outcomes. For instance, the subcutaneous ICD (S-ICD) has been developed comprising an active generator placed subcutaneously or inter-muscularly in the left hemithorax, connected to a defibrillation lead tunneled subcutaneously parallel to the left parasternal margin [3]. The S-ICD system provides similar efficacy in preventing sudden cardiac death compared to conventional ICDs while avoiding the well-known shortcomings of the transvenous leads [4]. Moreover, by precluding the heart and vessels, there is no related risk of systemic infections, and the associated infections are localized (pocket infection) and are less challenging to manage [5]. However, these devices can provide only transient percutaneous post-shock backup pacing and are unsuitable for patients with pacing indications. Moreover, the leadless PM (LPM) is a miniaturized capsule-like device, typically inserted through the femoral vein and affixed to the right ventricle myocardium using a dedicated active fixation mechanism [6]. These devices carry an extremely low risk of related infections, mainly by obviating the need for the device pocket, which is a major source of conventional CIED infections [7]. Furthermore, the smaller device surface, its extensive encapsulation, and the surrounding turbulent blood flow within the right ventricle may present additional protective factors in this regard [7]. Of note, an absorbable antibacterial mesh-like envelope has been utilized at the time of CIED implantation or replacement with promising results regarding the reduction of infections [8]. This approach may be considered particularly in patients with relapsing infections or who are at increased risk of future device infections [9].

Infective endocarditis (IE) is one of the most threatening CIED-related infections associated with high mortality rates and requires prompt detection and management [10,11]. Diagnosis is mainly based on the classical IE diagnostic criteria [12] as the presence of systemic infection symptoms/signs, bacteremia (positive blood cultures), characteristic echocardiographic features (vegetations or peri-valvular abscess), usually associated with at least one of the following elements: signs of CIED pocket infection, lead vegetations, positive cultures of the extracted lead materials, or abnormal metabolic activity at the level of CIED components (device or leads) revealed by advanced imaging tools (PET or SPECT) [13]. Transvenous lead extraction (TLE), combined with prolonged antibiotic therapy, has been validated as a valuable, effective approach in patients with CIED-related IE, even those with considerable lead vegetation [14]. Early extraction of the infected CIED system has been associated with improved clinical outcomes and is currently recommended by international societies’ guidelines [13,15]. However, adherence to the guidelines and TLE in patients with infected CIED still needs to improve in daily clinical practice [16]. Moreover, the in-hospital and long-term mortality is still relatively high following TLE for CIED-related systemic infection or IE emphasizing that prevention remains the essential step to accomplish [17,18].

While early and complete removal of the CIED system is strongly recommended for related IE or systemic infection, subsequent reimplantation still represents a clinical challenge, with many gaps in knowledge and current guidelines to manage these patients susceptible to related complications and infection relapses [19,20]. Based on the available literature data and the authors’ experience, this review aims to address the clinical and practical considerations of CIED reimplantation regarding careful indication reassessment, procedure timing, and the type/site of reimplanted CIED.

## 2. Decision-Making Elements

### 2.1. Clinical Assessment

Detailed clinical history and physical examination represent the essential first step for decision-making regarding CIED reimplantation.

Importantly, the awareness of risk factors or “red flags” associated with increased or recurrent CIED infections is essential to adopt appropriate preventive strategies, to keep a low threshold for diagnosis suspicion, and to optimize patients’ management. These risk factors are typically classified into patient-, procedure-, and device-related [21]. Comorbidities represent patient-related factors, including diabetes mellitus, renal insufficiency or dialysis, heart failure, chronic obstructive pulmonary disease, malignancy, and corticosteroids or anticoagulants use. On the other hand, procedure-related factors are redo-procedures comprising device replacement or reintervention for lead dislodgment, post-implantation pocket hematoma, temporary pacing, inexperienced operator, prolonged procedure duration, and lack of pre-procedural antibiotic prophylaxis. Finally, device-related factors are multiple leads, abdominal pocket, and epicardial leads [21]. 

Clinicians should focus on the original implant indication (e.g., primary vs. secondary ICD indication), the underlying structural or electrical heart disease, syncopal events before and following CIED implantation, arrhythmias occurrence and burden, heart failure and NYHA class, pharmacological therapy, comorbidities, patient’s preferences, and his life expected longevity and quality. Furthermore, the clinical entity of IE, the presence of vegetations, the bacterium type, and the clinical response to antibiotic therapy (body temperature, blood cultures and exams including WBC, ESR, CRP and procalcitonin) are also crucial to establishing the appropriate reimplantation timing.

### 2.2. CIED Interrogation 

Comprehensive device interrogation combined with previous reports can provide valuable data regarding pacing percentages and dependency, atrial or ventricular arrhythmias, and appropriate ICD therapies. It is reasonable to reprogram the device’s anti-brady parameters before lead extraction, aiming to favor the patient’s intrinsic rhythm and assess the actual pacing dependency. 

### 2.3. Peri-Procedure Rhythm Monitoring 

Management of patients with CIED-related IE often requires prolonged hospitalization. This should offer the opportunity for extended telemetry or Holter recording before and following TLE, providing a current assessment of the patient’s rhythm status, pacing needs and relevant arrhythmias.

### 2.4. Cardiovascular Imaging

Basal transthoracic echocardiography provides valuable information regarding left ventricular function and associated valvulopathy. Transesophageal echocardiography is more accurate in assessing the presence and size of leads or valve vegetations, which may guide or modify the lead extraction approach and impact the reimplantation timing [20,22]. Notably, if vegetation were visualized by cardiac imaging, CIED reimplantation should be delayed until blood cultures are negative for at least 2 weeks as recommended by the recent European Society of Cardiology guidelines [20].

Moreover, advanced imaging techniques such as PET or SPECT by revealing abnormal metabolic activity may add valuable information about the involvement of CIED components in the infection process or detect residual infection resources following TLE [2,13]. Finally, it is prudent to perform venous angiography and assess the patency of the contralateral axillary-subclavian venous axis to avoid unpleasant surprises at reimplantation and to allow better planning for potential alternative CIED options if possible (e.g., leadless or epicardial pacing). 

### 2.5. Electrophysiological Study (EPS)

Before deciding to abandon CIED reimplantation after TLE, an EPS might be considered in highly selected patients to assess ventricular tachyarrhythmias inducibility or localize the level of AV conduction delay (i.e., supra vs. infra-Hisian). For instance, EPS can be helpful after CIED extraction to stratify the arrhythmic risk and reassess ICD indication in a patient with ischemic cardiomyopathy who was initially implanted for primary prevention, with subsequent EF improvement (>35–40%) and documented non-sustained ventricular tachycardia (VT) [23]. Similarly, in patients who do not fulfill the primary prevention criteria, or were previously implanted for unclear indications, EPS may be considered to assess the risk of sustained VT in specific clinical conditions such as cardiac sarcoidosis, myotonic dystrophy and Tetralogy of Fallot [23,24]. Though controversial, programmed ventricular stimulation (single and double ventricular extrastimuli) might be considered for risk stratification in asymptomatic Brugada patients with spontaneous type I patterns [23], particularly before the abandonment of ICD reimplantation. Nevertheless, it is reasonable to postpone such an invasive diagnostic procedure until the complete resolution of the infection process.

## 3. Reassessment of CIED Indication

Redo CIED procedures carry a higher risk of infection complications [1]. This is even more relevant for patients who have already developed severe device-related infections such as IE, reflecting an intrinsic susceptibility. Therefore, the current CIED indication should be reassessed before TLE for better planning of the patient subsequent clinical course. If CIED implantation is not indicated anymore after a careful evaluation, reimplantation can be abandoned, keeping the patient under strict follow-up. In a retrospective single-center study including 150 patients who underwent TLE (23% for IE), about a third of patients did not undergo reimplantation without impacting survival during a 3.5-year follow-up period [25]. In another study including 854 patients undergoing TLE, mostly for infection indications, ~25% of patients did not require reimplantation initially. However, about a quarter of these patients did undergo reimplantation during a long follow-up period (40 months) highlighting the importance of close surveillance of patients who were not initially reimplanted [26]. Moreover, in a large retrospective study including 678 patients undergoing TLE, 14% of patients were not implanted and had reduced overall survival not related to major arrhythmias, which were rare in these patients [27].

Accordingly, it is imperative to reassess the actual indication for CIED reimplantation since the patient’s clinical condition and rhythm status may change following the first implantation. In daily practice, many clinical scenarios can be encountered concerning patients who may not need a CIED anymore:

- Complete recovery of AV conduction in PM patients implanted for early AV block following remote cardiac surgery or transcutaneous aortic valve implantation.
- Borderline or class IIb PM indications without subsequent clinical benefit.
- Elderly patients who underwent empirical PM implantation for syncope associated with bifascicular block or carotid hypersensitivity without benefit despite correct device functioning and near 0% pacing percentage at device interrogation.
- ICD patients, excluding CRT, for primary prevention with remarkable improvement in LV function (e.g., EF ~50%) and no ventricular tachyarrhythmias or appropriate ICD therapies in the device memory and previous follow-up.
- CRT-P patients who are either non-responders despite optimal LV lead site and pacing percentage or those who recovered the interventricular conduction delay (typically left bundle branch block) showing narrow intrinsic QRS and preserved AV conduction.
- Patients who refused reimplantation.

In summary, about 15 to 35% of patients do not undergo CIED reimplantation following TLE, mainly because they do not meet implantation indications anymore, without direct impact on cardiovascular mortality if carefully selected [25,26,27]. Therefore, CIED indication reassessment is essential to avoid, when possible, reimplantation in these vulnerable patients who already developed threatening CIED infections such as IE. However, non-reimplanted patients should be educated regarding alarming symptoms (e.g., pre-syncope, progressive dyspnoea) and kept under close surveillance including periodic clinical assessment and prolonged periodic Holter recordings. 

If continuous rhythm monitoring is desired, implantable loop recorders (ILR) can be considered. ILR provides the possibility of daily-basis follow-up and early detection of potential arrhythmias, which may necessitate CIED reimplantation. This strategy may be adopted in patients with doubtful or unclear device indications, such as asymptomatic Brugada patients with spontaneous type I pattern without documented or induced ventricular arrhythmias and those with a bifascicular block with unclear symptoms and no previous recording of advanced or complete AV block.

## 4. Timing of CIED Reimplantation

Once CIED indication is reconfirmed following TLE, the next challenge is establishing the appropriate timing for device reimplantation. While this is generally not a concern following TLE for non-infection causes (e.g., lead failure) since reimplantation can be accomplished even during the same procedure at the ipsilateral side, reimplantation timing following TLE in patients with IE can represent a clinical dilemma. In the absence of solid prospective literature data, reimplantation timing should be individualized and based on a careful risk-benefit assessment of each patient. 

A balance is warranted between early reimplantation which may be associated with a high risk of infection relapse, and delayed reimplantation which may require prolonged temporary pacing, hospitalization, and ICU stay with substantial associated risks and health system costs. In one retrospective single-center study including 109 patients who underwent TLE for related IE, the 1-year mortality rate was remarkably higher in those reimplanted < 14 days following CIED removal (27% vs. 8%), particularly in the presence of vegetations (41%) [28]. When practicable, it is desired to postpone reimplantation 4 or 6 weeks after TLE, thus allowing a full course of IE antibiotic therapy and complete healing of the infection process. On the other hand, in patients who need immediate CIED functions, CIED can be reimplanted at the contralateral side after at least 72 h of persistent negative blood cultures and in the absence of vegetations [13,15,20].

However, “bridging” therapy allows for delayed reimplantation even in these patients (i.e., pacing-dependent).

Elements favoring delayed reimplantation strategy include the absence of immediate need for CIED functions, vegetations, residual unextracted CIED materials “ghosts” or other potential sources of infection, and delayed clinical response to antibiotic therapy such as persistent fever, bacteremia, or multi-resistant IE bacterium. On the other hand, early implantation is desired in pacing-dependent patients, particularly those who show accelerated clinical response to antibiotic therapy (afebrile, negative blood cultures). Moreover, prolonged periods of device lack in CRT responders may be associated with worse clinical outcomes [29]. Plausibly, patient eligibility for a different CIED type with lower infection risks (S-ICD or leadless PM) may facilitate early reimplantation in some patients. Cardiac imaging as repeated TEE to assess response to therapy and nuclear imaging in case of suspected septic emboli may also guide reimplantation timing. 

**Figure 1:**
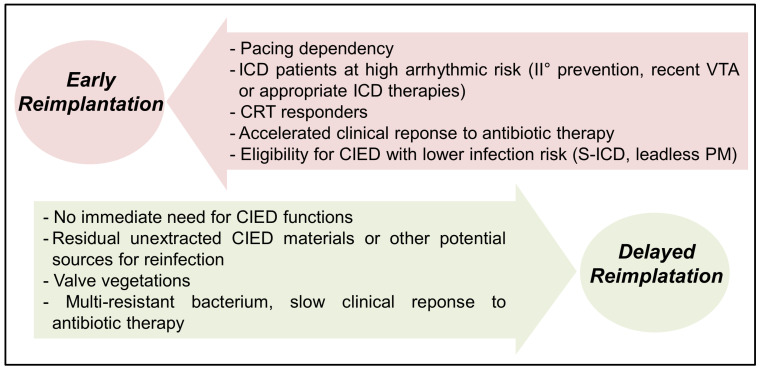
Elements favoring early vs. delayed CIED reimplantation after TLE for IE. VTA: ventricular tachyarrhythmias; CRT: cardiac resynchronization therapy; CIED: cardiac implantable electronic device; ICD: implantable cardioverter-defibrillator; PM: pacemaker; S-ICD: subcutaneous ICD.

Finally, reimplantation timing should be based on a multidisciplinary team evaluation, including clinical cardiologists, electrophysiologists, infectious disease specialists, cardiac surgeons and cardiac imaging experts. Figure 1 summarizes the clinical elements favoring early versus delayed CIED reimplantation following extraction for CIED-related IE.

## 5. The Type and Location of Reimplanted CIED

Once the indication of CIED is reconfirmed and the appropriate reimplantation timing is established, the new CIED system is typically implanted on the contralateral side to avoid close contact with the previously infected region. Considering the intrinsic infection risk in patients with recent IE, it is reasonable to consider implanting a different CIED type less prone to systemic infection if clinically applicable. This approach may also shorten the waiting period between extraction and reimplantation when desired. 

### 5.1. Subcutaneous Defibrillator 

The S-ICD offers a valuable alternative in patients following TLE for systemic infection or IE who do not require pacing therapies (anti-bradycardia, anti-tachycardia or CRT) [30]. The S-ICD nature, having no contact with the endovascular system or bloodstream, eliminates the risk of related IE as confirmed in long-term data of the EFFORTLESS registry including 984 patients with 5-year follow-up [31]. Accordingly, the S-ICD can be reimplanted early following infected CIED extraction. However, and until further evidence is available, a waiting period of 72 h following negative blood cultures is advisable and is usually feasible in daily practice. 

### 5.2. Leadless Pacing

The LPM is an evolving technology with apparently reduced risks of related infections, likely by obviating the need for the device pocket/incision as a potential factor in the infection process. Therefore, the leadless PM has been adopted in recent studies following TLE of infected CIED, or even during the same extraction procedure, with promising results regarding infection relapses [32,33]. However, concomitant LPM implantation during CIED extraction for active IE cannot be recommended based on these data since the LPM itself is not completely immune to infections, and a few cases of related IE with device vegetation have been reported [34]. Accordingly, we believe that it is prudent to postpone LPM implantation for at least 72 h following negative blood cultures, and preferably for 2 weeks if vegetations were visualized. 

### 5.3. Epicardial Pacing

Theoretically, a pacing system positioned within the pericardium without direct contact with the bloodstream might be less exposed to infection, especially in patients with recent or active bacteremia. This approach has been adopted effectively in some hemodialysis patients who underwent removal of their infected transvenous PM with subsequent epicardial lead implantation [35]. A surgical approach combining lead extraction and epicardial PM placement has been performed in some studies to manage pacing-dependent patients requiring CIED extraction for related IE to accelerate patient mobility and shorten hospital stay. However, a recent meta-analysis including 339 pacing-dependent patients comparing prolonged temporary pacing plus delayed endocardial CIED reimplantation to early surgical implantation of epicardial PM showed that the latter strategy shortened hospitalization but was associated with worse clinical outcomes regarding mortality, late infections and CIED redo-interventions [36]. Therefore, the surgical epicardial approach should be limited to pacing-dependent patients who have other surgical indications, being managed in centers with a highly-experienced surgical team, or where prolonged hospitalization is not accepted by the patient or local health system.

### 5.4. Bridge Therapy

This approach should be considered in patients who are highly dependent on their CIED functions but whose infection status of active IE does not allow early (i.e., 3 to 5 days) device reimplantation, particularly if there is a slow response to antibiotic therapy, persistent fever or bacteremia, or residual vegetations. For pacing-dependent patients, bridge pacing is typically provided via an active fixation lead introduced percutaneously through the ipsilateral internal jugular, or subclavian (by recapturing the previous access), vein to the right ventricle and connected to an externalized (reused) pacemaker device secured on the patient’s chest. This bridge pacing technique allows stable prolonged (e.g., several weeks) temporary pacing until CIED reimplantation can be safely performed at the contralateral side with a low risk of related complications [37,38,39]. Furthermore, it allows early patient mobility and monitoring in the cardiology department rather than the intensive care unit, while some selected patients could be discharged home or to a nurse facility. 

On the other hand, patients requiring defibrillation therapy protection for their high-risk profile (secondary prevention, recent ventricular tachyarrhythmias or appropriate ICD therapies) may undergo implantation of an externalized ICD system by placing an active fixation dual-coil defibrillation lead in the right ventricle through the ipsilateral subclavian vein and programming the shock configuration between the two coils (passive can modality) [40]. Dell’Era et al. reported their experience in 18 patients who were effectively managed by adopting this approach without relevant related complications [41]. In the latter study, the mean duration of the externalized ICD was 16.5 (4–30) days before definitive ICD implantation, and one patient experienced arrhythmic storm that was successfully detected and treated by anti-tachycardia pacing and shocks delivered from the temporary externalized ICD system [41]. 

Finally, patients who require only ICD protection without immediate pacing needs may benefit from the wearable cardioverter-defibrillator as a bridge until their clinical condition and infection status allow for reimplantation of a definitive ICD. In a large retrospective study including 8058 patients who utilized this wearable device following ICD removal for related infection, the risk of ventricular tachyarrhythmias was higher in the first two months (4%), and the device showed high efficacy in treating these arrhythmias while the incidence of inappropriate shocks was relatively low (2%) [42]. However, patient compliance, availability and costs of this technology may vary widely with considerable limitations in daily clinical practice.

## 6. Special Considerations for CIED Reimplantation

### 6.1. CRT Patients

As mentioned earlier, it is advisable to avoid prolonged periods (>14 days) of ventricular dyssynchrony in CRT responders since their heart failure may precipitate [29]. Furthermore, once the CRT indication is reconfirmed, a few technical aspects should be considered when planning the reimplantation procedure. CRT implantation at the contralateral side (i.e., from the right side) can add some technical difficulties during left ventricular lead positioning. More importantly, CRT reimplantation following CIED extraction has a lower success rate, likely due to coronary sinus or its branches stenosis/occlusion and may require unconventional techniques [43]. Moreover, CRT reimplantation is associated with higher infection rates compared to the first implantation, emphasizing the importance of CRT indication reassessment in these patients [43,44]. Finally, His or left bundle pacing may be considered as an alternative option in CRT-P patients aiming to reduce the lead number and overcome the abovementioned technical difficulties associated with redo-CRT using the coronary sinus branches. However, data regarding the feasibility and safety of extracting chronic left bundle pacing leads are still limited and further research is needed [45]. 

### 6.2. Cardiac Surgery Evaluation

Patients with CIED-related IE should have an early multidisciplinary evaluation (cardiac surgeon, cardiologist, infectious disease specialist, cardiac imaging expert) for potential surgical indications such as IE complicated with heart failure, valvular dysfunction, peri-valvular abscess, relapsing bacteremia despite antibiotic therapy, and extremely large or mobile vegetations with eminent embolic risk [12,46]. These patients are unsuitable for TLE and are candidates for cardiac surgery to treat IE, extract the CIED system, and implant epicardial leads (for PM patients) after a comprehensive assessment of the peri-operative risks and detailed discussion with the patient. 

### 6.3. Cardiac Magnetic Resonance (CMR)

Though CMR can be performed safely under strict control in most patients with CIEDs, even those with older devices (non-MRI conditional), the exam might be limited by organizational aspects, medical staff resources, and local institute protocols. More importantly, image quality, diagnostic utility and clinical interpretability of CMR can be substantially limited by artifacts in CIED patients (particularly ICD) [47,48]. The CIED system removal occasionally offers a unique opportunity to perform a complete diagnostic CMR that may be essential in future patient management. For instance, an ICD patient with a previous “borderline or doubted” diagnosis of arrhythmogenic right ventricular dysplasia who underwent TLE for IE may benefit from a new diagnostic CMR that can guide clinical management or the decision of reimplantation. Similarly, a new CMR can be performed in a patient with idiopathic or ischemic cardiomyopathy following device extraction to accurately assess the burden and distribution of myocardial scar, providing valuable data if catheter ablation of ventricular arrhythmias should be planned during follow-up. This diagnostic exam can be performed in relatively stable patients following IE where CIED reimplantation is not indicated anymore or can be at least deferred without bridge therapy.

### 6.4. Medical-Legal Issues

In the case of no-reimplantation, these issues might arise if a hazardous clinical outcome (e.g., cardiac arrest) should occur following hospital discharge. Such concern must not affect a clinician’s decisions that should be based on in-depth clinical assessment and analysis of the risk-benefit balance in an individual patient. However, it is still prudent to document complete medical files supporting the decision not to reimplant based on a multidisciplinary team evaluation, rather than a single physician decision, involving the cardiac electrophysiologist, the reference cardiologist, and the infectious disease specialist. Moreover, it is vital to comprehensively inform the patient and his family of this decision rationale and the related risk-benefit.

### 6.5. Reimbursement Issues

Lead extraction procedures often require prolonged hospitalization, ICU stay, cardiac surgery standby, and advanced technologies with considerable related costs that are not well reimbursed by many health systems worldwide [49]. Moreover, CIED reimplantation aggravates the economic impact, particularly during the index hospitalization, with subsequent concerns regarding local reimbursement policies. Though this aspect should not affect the clinical decision of CIED reimplantation or its timing, regional and national health authorities must adopt appropriate measures to overcome this issue and optimize patient management.

**Figure 2:**
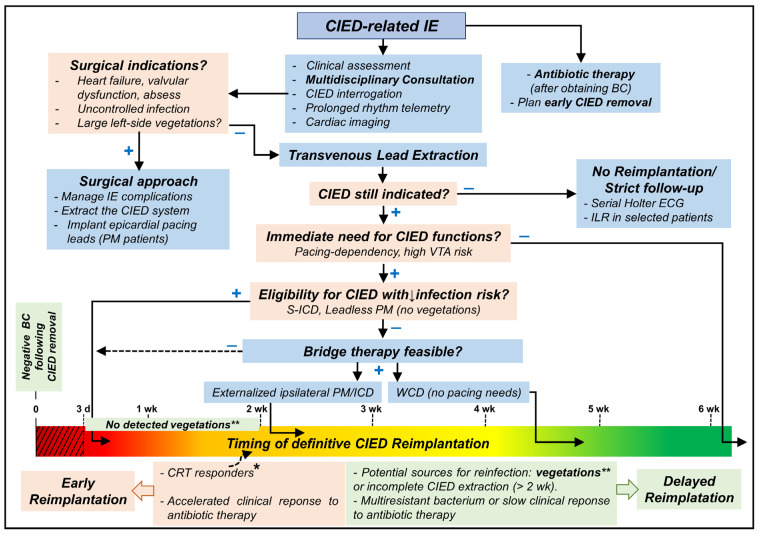
Central Illustration showing a proposed approach to manage patients undergoing lead extraction for CIED-related IE. VTA: ventricular tachyarrhythmias; CRT: cardiac resynchronization therapy; CIED: cardiac implantable electronic device; ICD: implantable cardioverter-defibrillator; PM: pacemaker; S-ICD: subcutaneous ICD; IE: infective endocarditis; BC: blood culture(s); ILR: implantable loop recorder; WCD: wearable cardioverter-defibrillator; d: day; wk: week; +: yes; −: no. CIED reimplantation should be avoided when possible for at least 72 h of negative blood culture (red dotted area). * indicates some patients who may be reimplanted earlier (10–14 days after negative blood cultures) as CRT responders or those with bridge-therapy who showed favorable clinical response or when delayed reimplantation is impractical for the patient or local health institute. ** The presence of vegetation should delay reimplantation for at least 2 weeks after negative blood cultures; the only exception might be S-ICD implantation.

The Central Illustration (Figure 2) shows a proposed approach to manage patients undergoing lead extraction for CIED-related IE.

## 7. Conclusions

CIED reimplantation following lead extraction in patients with related IE presents a clinical challenge in daily practice. A careful reassessment of CIED indication, procedure timing, and the reimplanted CIED type/site is essential. A tailored, multidisciplinary approach involving clinical cardiologists, electrophysiologists, cardiac imaging experts, cardiac surgeons, and infectious disease specialists is crucial to optimize these patients’ management and clinical outcomes. While prevention is still the most important early step, further studies are required to refine the best clinical approach to manage patients with CIED-related IE following system removal.

## Data Availability

Data are available in the references list and further information can be provided via a reasonable request to the corresponding author.
